# Purchasing medicines and functional foods on the internet: a cross-sectional study investigating the knowledge, attitudes, and experience of Vietnamese people in 2023

**DOI:** 10.1186/s12889-024-20103-w

**Published:** 2024-09-27

**Authors:** Dung Anh Doan, Nhung Hong Vu, Phuong Lan Nguyen, An Duc Nguyen, Dai Xuan Dinh

**Affiliations:** 1 Faculty of Pharmacy, Phenikaa University, Hanoi, Vietnam; 2 Faculty of Pharmaceutical Management and Economics, Hanoi University of Pharmacy, Hanoi, Vietnam; 3 Drug Administration of Vietnam, Ministry of Health, Hanoi, Vietnam

**Keywords:** Online shopping, Online purchasing, Online buying, Medicine, Dietary supplement, Functional food, Medical product, Internet, Vietnam

## Abstract

**Objective:**

To investigate Vietnamese people’s knowledge, attitudes, and experience in purchasing medicines and functional foods online.

**Methods:**

Via an online survey, the data of 1,070 participants were collected, including their general characteristics, Internet use, previous experience, knowledge, and attitudes towards purchasing medicines/functional foods online. Factors associated with their knowledge and attitudes were identified via multivariate linear regression models.

**Results:**

During 2022–2023, about 97.2% of participants used the Internet to seek health information (self-diagnosis: 65.0%, self-medication: 72.6%). Roughly 52.8% bought medicines and/or functional foods online. Among 565 buyers, 41.8% felt satisfied. Only 19.9% understood that selling medicines online was illegal in Vietnam. The main benefits of purchasing medicines/functional foods online that many people agreed on included convenience (87.1%), freedom from location (84.8%), and being able to order/buy products after opening hours (84.7%). Many people felt worried about the ability to buy counterfeit or substandard products (87.7%), inaccurate product information (85.0%), the lack of supervision of the authorities (83.7%), and increasing risks of drug abuse, self-medication, and treatment non-adherence (82.5%). Roughly 84.3% found distinguishing between legal and illegal online pharmacies difficult. Participants’ average knowledge and attitude scores were 6.514 ± 2.461 (range: 0–16) and 89.330 ± 13.720 (range: 23–115), respectively. The main factors associated with people’s knowledge and attitudes towards purchasing these products online included their frequency of Internet use, seeking health information online for self-medication, feeling satisfied with previous experience, and having at least one chronic disease.

**Conclusions:**

Many Vietnamese people’s knowledge about purchasing medicines/functional foods online was limited. With the increasing need for online shopping, enhancing their knowledge is paramount. In the forthcoming years, when the Ministry of Health and relevant authorities publish legal documents and enact laws involving online pharmacies and trading medicines on the Internet, ways to recognize licensed online pharmacies must be widely propagated and disseminated in the community.

**Supplementary Information:**

The online version contains supplementary material available at 10.1186/s12889-024-20103-w.

## Background

The development of digital technology and the omnipresence of electronic gadgets have ushered in the golden era of globalization. The global internet boom can give rise to both benefits and drawbacks. In recent years, the Internet and social media have played many essential roles in the healthcare and medical sectors, such as Internet-based lifestyle counseling (e-counseling) [1]. The Internet has become an easy way for people to buy goods and services, including health products. Easy access to the Internet helps people to seek and retrieve health information. High proportions of people using the Internet to seek health information have been reported in many countries, for example, 84.8% in Germany (2017) [2], 75.1% in the United States (2017) [3], 77.1% in China (2018) [4], and 77.0% in Australia (2021) [5]. In the United States, web-based prescription-filling behavior among adults significantly increased from 5.9% in 2009 to 11.5% in 2018 [6]. However, the appearance and development of both legal and illegal online pharmacies (e-pharmacies) have engendered numerous controversies. Besides benefits such as improving access to medicines, many grave concerns are associated with trading medicines on the Internet, such as selling prescription-only medicines without a prescription, counterfeit medicines, and consumer fraud [7]. Illegitimate online pharmacies have been a matter of concern. Findings from a study conducted in 2020 showed that of 89 online pharmacies selling COVID-19 treatments, only seven pharmacies (7.9%) were legitimate. Sixty-two pharmacies (69.7%) were rogue, and 20 pharmacies (22.5%) were unapproved [8].

Numerous factors have facilitated the growing market for selling and buying health products online (such as the rapid expansion of the Internet, convenience, and customer satisfaction with online shopping). In Vietnam, it is estimated that 72.1 million people are using the Internet (72.4% of the entire population) [9]. A study conducted in 2015 with the participation of 1,080 young Vietnamese citizens showed that 72.9% of them were interested in health information on Facebook. About 17.5% presumed that information received from this social network was valuable and reliable [10]. During the COVID-19 pandemic, lockdowns and self-isolation have been additional rationales behind the increase in online shopping among people worldwide. The results from previous studies showed that the proportion of people buying health products over the Internet has been relatively high, especially during the COVID-19 pandemic (for example, 8.3% (Romania, 2010–2011) [11], 8.4% (Hungary, 2010–2011) [12], 31.2% (United Arab Emirates, 2020) [13], and 36.5% (Saudi Arabia, 2020) [14]). However, in many countries (including Vietnam), regulatory frameworks specific to e-pharmacy and online sales of medicines have yet to be enacted [7]. In Vietnam, it is illegal to sell medicines via the Internet. One problem with e-pharmacies was that people found it difficult to distinguish between legitimate online pharmacies and rogue/unapproved websites [8]. To guarantee the rational use of medicines and other health products, investigating the general population’s knowledge, attitudes, and practices (KAP) regarding the online purchase of these products is paramount. Only a negligible number of studies involving this topic were conducted [14–23]. This study aimed to investigate Vietnamese people’s knowledge, attitudes, and experience regarding purchasing medicines and functional foods online.

## Methods

### The questionnaire

A set of questions (the initial questionnaire) was drafted and developed for this study based on a literature review of many previous studies [11–24]. Two lecturers (from the Hanoi University of Pharmacy and Phenikaa University) and one official (from the Drug Administration of Vietnam, Ministry of Health) reviewed the suitability of questions (face validity). Then, a pilot study was conducted with the participation of 30 people to guarantee that questions were clear and intelligible. Two weeks later, these people answered the knowledge and attitude parts of this questionnaire one more time. The final questionnaire consisted of three main sections (Supplementary Material 1). The first section was 12 questions about participants’ general characteristics (such as year of birth and education level). The second section included five questions about participants’ Internet usage and seven about their previous experience in purchasing medicines/functional foods online. The last section encompassed eight knowledge questions and 23 attitude questions regarding purchasing medicines/functional foods online (5-point Likert scale [25]). Medicine, functional food, and online shopping were defined in the second section to ensure respondents could comprehend the questions.

The reliability of this questionnaire (for the knowledge and attitude parts) was assessed via Cronbach’s alpha, McDonald’s Omega, Spearman-Brown split-half reliability, and intraclass correlation coefficients (ICCs). With the data from the pilot study, the good internal consistency of this questionnaire was demonstrated via Cronbach’s alpha (knowledge part: 0.850, attitude part: 0.950) and Omega Total (0.960). In addition, the Spearman-Brown split-half reliability was 0.822 for the knowledge part and 0.936 for the attitude part. The test-retest reliability was evaluated between two times of measurements (two weeks). Participants’ average scores slightly increased, but the differences were insignificant (knowledge scores: 8.500 and 8.833, *p* = 0.700; attitude scores: 98.533 and 98.767, *p* = 0.915). ICCs higher than 0.750 (knowledge part: 0.801 (95%CI: 0.668–0.893), attitude part: 0.934 (95%CI: 0.895–0.964)) also demonstrated the good test-retest reliability of this questionnaire when it was used to collect data at two different times of measurement.

### Study setting, sampling method, and data collection

To have a margin of error of 5%, a confidence level of 99%, and a response distribution of 50%, the minimum sample size was 664 people (Raosoft sample size calculator). We strived to approach as many Vietnamese people as possible to increase the generalization and extrapolation of findings. Eligible participants were those aged 18 and older, able to understand Vietnamese, using the Internet, and willing to participate in this research. The participants were recruited via convenience and snowballing sampling methods (non-random sampling techniques). From March 09 to May 11, 2023, the questionnaire (designed with Google Forms) was sent to participants through online platforms and social networks (such as Facebook, Messenger, Zalo, emails, or using quick response codes). We also encouraged the participants to send our questionnaire to their friends, colleagues, and relatives. The answers of 1,082 Vietnamese people were recorded. However, 12 people said that they did not agree to participate in this research. Finally, 1,070 people (98.9%) were included in the data analyses.

### Data analysis

The knowledge and attitude sum scores were computed for each participant. For eight knowledge questions, 0 scores were given for incorrect answers, while 1 and 2 scores were assigned for correct and totally correct answers, respectively. The knowledge sum score of one person ranged from 0 to 16. For 23 questions involving people’s attitudes towards the potential advantages and disadvantages of purchasing medicines/functional foods online, the score of a person varied from 1 (totally disagree) to 5 (totally agree). The attitude sum score of one person ranged from 23 to 115. Higher scores indicated better knowledge and more positive attitudes towards purchasing medicines/functional foods online. A 50% cut-off point (high: ≥50%, low: <50%) and Bloom’s cut-off point (high: ≥80%, moderate: 60% to < 80%, low: <60%) were used to categorize the knowledge and attitude scores of participants, similar to many previous KAP studies [26–29].

Data were analyzed using R software (version 4.4.0). To describe the general characteristics and Internet usage of participants, numbers (percentages) and means (standard deviations) were used (respectively for categorical and numeric variables). Data normality was checked using histograms and the Shapiro-Wilk test (a *p*-value > 0.05 indicating a normally distributed continuous variable). Factors associated with people’s knowledge and attitudes towards the online purchase of medicines/functional foods (sum scores) were first identified via univariate linear regression models. To minimize the complexity of models and prevent multicollinearity and overfitting, we selected independent variables in two multivariate linear regression models using the Bayesian Model Averaging method. Multicollinearity was checked via the variance inflation factor (VIF). A *p*-value lower than 0.05 denoted statistical significance.

## Results

### General characteristics of participants

A majority of the participants were aged under 45 years old (85.7%). Nearly two-thirds (65.2%) were females and 47.2% got married. Nearly three-fifths were living in northern Vietnam (59.0%). Most of the participants (80.3%) came from urban areas. Their highest education levels were high school (48.8%) and university (33.1%). Approximately 6.7% (72 people) were working in the medical field. On average, the monthly income/allowance/retirement pension of 672 people (62.8%) was less than 9 million Vietnam dongs (equivalent to 379.747 US dollars). Only a negligible number of participants have no health insurance cards (2.6%). A fifth (19.6%) had at least one chronic disease (for example, diabetes mellitus, hypertension, sinusitis, chronic obstructive pulmonary disease, and bronchial asthma) (Table 1).

**Table 1:** Main characteristics of the participants (*n* = 1,070 Vietnamese people)

No	Characteristics		Number	%
1	Sex	Male	372	34.8
		Female	698	65.2
2	Age (years old)	19 to 24	498	46.5
		25 to 34	199	18.6
		35 to 44	220	20.6
		45 or above	153	14.3
3	Marital status	Married	505	47.2
		Unmarried	565	52.8
4	Region	Northern	631	59.0
		Central	280	26.2
		Southern	159	14.9
5	Area	Urban	859	80.3
		Rural	211	19.7
6	Highest level of education	Secondary school or lower	35	3.3
		High school	522	48.8
		College and intermediate	115	10.7
		University	354	33.1
		Post-university (Master, Ph.D…)	44	4.1
7	Occupation	Healthcare jobs	72	6.7
		Non-healthcare jobs, students, retired	998	93.3
8	Average monthly income, allowance, or retirement pension (mVND*)	< 3	333	31.1
		3 to < 6	199	18.6
		6 to < 9	140	13.1
		9 to < 12	81	7.6
		12 to < 15	66	6.2
		15 to < 18	77	7.2
		18 or more	174	16.3
9	Health insurance	Yes	1,042	97.4
		No	28	2.6
10	Having at least one chronic disease	Yes	210	19.6
		No	860	80.4

*: 1 mVND (million Vietnam dongs) = 49.194 US$

## Internet use and purchasing medicines/functional foods online in the past year

During 2022–2023, nearly three-quarters of participants usually used the Internet (70.2%). About 12.5% (134 people) had a habit of purchasing products online more than three times per week. Almost all participants (97.2%) used the Internet to seek health information, including 696 cases of self-diagnosis without visiting a doctor (65.0%) and 777 people self-medicating their health issues based on health information seeking on the Internet (72.6%). Over half (52.8%) bought medicines and/or functional foods online. Roughly 14.6% (156 people) purchased these products from foreign sources on the Internet. Only 189 participants (17.7%) purchased medicines and/or functional foods on the Internet more than three times in the past year. Among 565 people who had previous online purchasing experience, approximately two-fifths (41.8%) felt satisfied (Table 2).

**Table 2:** Internet use and purchasing medicines/functional foods online among the participants in the past year (*n* = 1, 070 Vietnamese people)

No	Characteristics of Internet use and online shopping		Number	%
1	The prevalence of Internet usage	Rarely	62	5.8
		Sometimes	257	24.0
		Usually	751	70.2
2	The prevalence of online shopping (any products or services)	Rarely/never	588	55.0
		Sometimes	348	32.5
		Usually	134	12.5
3	Using the Internet to seek health information	Yes	1,040	97.2
		No	30	2.8
4	Self-diagnosis without visiting a doctor based on health information from the Internet	Yes	696	65.0
		No	374	35.0
5	Self-medication without consulting a pharmacist or doctor based on information from the Internet	Yes	777	72.6
		No	293	27.4
8	Purchased medicines and/or functional foods on the Internet in the past year	Yes	565	52.8
		No	505	47.2
9	Purchased medicines and/or functional foods online from foreign sources (such as international websites)	Yes	156	14.6
		No	914	85.4
10	Purchased medicines and/or functional foods on the Internet in the past year (times)	No	505	47.2
		1 to 3 times	376	35.1
		> 3 times	189	17.7
11	Level of satisfaction for experience in the online purchasing of medicines and/or functional foods	Never	505	47.2
		Completely unsatisfied	11	1.0
		Unsatisfied	20	1.9
		Neutral/Normal	298	27.9
		Satisfied	161	15.0
		Completely satisfied	75	7.0

### Participants’ knowledge and attitudes towards purchasing medicines/functional foods on the internet

Of 1,070 participants, 88.8% thought checking the information of sellers/providers before online buying was necessary. About four-fifths presumed that before purchasing medicines on the Internet (especially prescription-only medicines), it was necessary to consult healthcare professionals (81.2%). However, less than half knew that nearly every medicine and functional food could be purchased over the Internet (48.9%). More importantly, only 213 people understood that selling medicines online was illegal in Vietnam (19.9%) (Table 3). Overall, the average knowledge score of participants was 6.514 ± 2.461 (median: 6, interquartile range: 5–8, min: 0, max: 16). The percentage of participants having high knowledge scores was low. About 30.2% (323 people) had a knowledge score of at least 8 (50% cut-off point), whereas only 12 people (1.1%) had a score of 12.8 or higher (Bloom’s cut-off point).

**Table 3:** The knowledge of the participants regarding purchasing medicines and functional foods on the internet (*n* = 1, 070 Vietnamese people)

No	Knowledge question (true statement)	Correct answers (%)
1	It is necessary to check the information (such as qualification and reputation) of the sellers/providers before online buying.	950 (88.8)
2	It is necessary to consult with healthcare professionals (such as doctors and pharmacists) before buying medicines online, especially prescription-only medicines.	869 (81.2)
3	Trading medicines online can go beyond the scope of a country, causing many difficulties and challenges in management.	725 (67.8)
4	Medicines and functional foods can be purchased on the Internet (online).	676 (63.2)
5	There are other reputable sources to buy medicines and functional foods besides pharmacies in the hospitals and the community.	674 (63.0)
6	Purchasing prescription-only medicines online without a prescription is illegal and inappropriate.	609 (56.9)
7	Nearly every medicine and functional food can be purchased on the Internet.	523 (48.9)
8	Selling medicines online is illegal in Vietnam.	213 (19.9)

Regarding people’s attitudes towards the online purchase of medicines/functional foods, the main advantages that many people chose included convenience (932 people, 87.1%), freedom from location (907 people, 84.8%), the ability to order and buy products after the opening hours (906 people, 84.7%), and easy to check products’ availability (894 people, 83.6%). The main disadvantages of online purchasing included the ability to buy expired, counterfeit, or substandard products (938 people, 87.7%); inaccurate information about products (910 people, 85.0%); lack of supervision of the authorities (896 people, 83.7%); increasing the risks of drug abuse, self-medication, and treatment non-adherence (883 people, 82.5%); and increasing the risks of polypharmacy and drug interactions (878 people, 82.1%). In addition, 902 people (84.3%) found it difficult to distinguish between legal online pharmacies and illegal commercial websites. In general, the average attitude score of all participants was 89.330 ± 13.720 (median: 90, interquartile range: 83–98, min: 23, max: 115). The percentage of participants having positive attitudes towards purchasing medicines/functional foods online was relatively high. About 1,016 participants (95.0%) had attitude scores of at least 69 (50% cut-off point) and 291 people (27.2%) had attitude scores of 96.6 or higher (Bloom’s cut-off point) (Table 4).

**Table 4:** The attitudes of the participants towards the potential advantages and disadvantages of purchasing medicines/functional foods on the internet (*n* = 1, 070 Vietnamese people)

No	The attitude	Mean score ± SD
A	Potential advantages	
1	Convenience (for example, doorstep delivery; in conditions of bad weather, pandemics, or epidemics; elimination of barriers for people with disabilities and senior citizens)	4.101 ± 0.802
2	Able to order and buy products after the opening hours (available 24/7)	4.012 ± 0.831
3	Easily check the availability of products (reduce the time spent traveling between pharmacies to check)	4.006 ± 0.865
4	Freedom from location (can purchase products from other countries)	4.006 ± 0.802
5	No waiting time or queuing at pharmacies	4.000 ± 0.875
6	When buying online, it is faster and easier to compare the information and prices of products than when directly buying in pharmacies	3.998 ± 0.805
7	Affordable, lower prices, many offers or discounts	3.721 ± 0.890
8	Wide range of products (can buy products unavailable or not sold at nearby local hospitals or community pharmacies)	3.700 ± 0.929
9	More privacy and anonymity	3.621 ± 1.008
10	In comparison with buying products in pharmacies, I can get more information when buying online	3.193 ± 1.100
**B**	**Potential disadvantages**	
11	Can purchase expired, counterfeit, and/or substandard products	4.121 ± 0.817
12	Difficult to distinguish between legal (registered) online pharmacies and illegal (unlicensed) commercial websites	4.087 ± 0.877
13	Information about products on the Internet may be inaccurate	4.056 ± 0.860
14	Lack of supervision of the authorities	4.056 ± 0.849
15	Increasing the risks of drug abuse, self-medication, and treatment non-adherence	4.051 ± 0.874
16	Increasing the risks of polypharmacy and drug interactions	4.049 ± 0.850
17	The sellers may not be precisely identified (names, qualifications…)	3.966 ± 0.899
18	People under 18 years old can purchase medications without restrictions	3.913 ± 1.007
19	Increasing the risks of personal information leakage and money transactions	3.869 ± 0.927
20	Products can be stored/preserved under substandard conditions	3.849 ± 0.888
21	Not getting the right products (mistakes in packing or delivery)	3.830 ± 0.886
22	Difficult to choose the appropriate product due to the great number of products on the Internet	3.631 ± 0.991
23	Long delivery time	3.495 ± 0.987

SD: standard deviation

A higher score means more positive attitudes towards purchasing medicines/functional foods online

As per the results of multivariate linear regression models, having at least one chronic disease (*p = 0.002*, *p < 0.001*), usually using the Internet (*p = 0.001*, *p < 0.001*), self-medication based on health information sought online (*p < 0.001*, *p = 0.005*), and feeling satisfied with previous experience (*p < 0.001*, *p < 0.001*) were four factors associated with Vietnamese people’s knowledge and attitudes towards the online purchase of medicines/functional foods, respectively. In addition, people living in the northern part had lower knowledge scores than those residing in the central and southern parts of Vietnam (*p < 0.001*). Education level (university: *p < 0.001*) and using the Internet for self-diagnosis (*p = 0.001*) were two additional factors associated with Vietnamese people’s attitudes (Table 5). Besides, according to univariate linear regression models, other factors that can be associated with people’s knowledge encompassed their education level (post-university: *p < 0.001*), marital status (*p = 0.003*), usually shopping online (*p < 0.001*), using the Internet for self-diagnosis (*p < 0.001*), and purchased medicines/functional foods in the past year (*p = 0.002*). Other factors associated with people’s attitudes consisted of monthly income/allowance (*p < 0.001*) and usually shopping online (*p < 0.001*) (Supplementary Material 2). Furthermore, there was a positive association between people’s knowledge and attitude scores (*p < 0.001*).

**Table 5:** Factors associated with the knowledge and attitudes of the participants regarding purchasing medicines/functional foods on the internet (*n* = 1, 070 Vietnamese people)

Independent variables	Knowledge		Attitude	
	acoef	*p*-value	acoef	*p*-value
1. Region (ref: Central and Southern)				
Northern	-0.643	< 0.001		
2. Highest level of education (ref: Others)				
University			-2.871	< 0.001
3. Having at least one chronic disease (ref: No)	0.576	0.002	5.678	< 0.001
4. Frequency of Internet use (ref: Rarely)				
Sometimes			6.851	< 0.001
Usually	0.525	0.001	7.250	< 0.001
5. Used the Internet for self-diagnosis (ref: No)			3.062	0.001
6. Used the Internet for self-medication (ref: No)	0.857	< 0.001	2.899	0.005
7. Satisfied with previous experience in purchasing medicines/functional foods online (ref: No)	1.355	< 0.001	3.774	< 0.001
Adjusted R-squared	0.139		0.108	

acoef: adjusted coefficient, ref: reference

In both multivariate linear regression models, VIFs of all independent variables were lower than 1.400

## Discussion

This study was carried out to investigate Vietnamese people’s knowledge, attitudes, and experience in purchasing medicines/functional foods on the Internet. The results showed that self-diagnosis and self-medication based on health information on the Internet were found among roughly two-thirds of participants. During 2022–2023, over half of participants purchased medicines and/or functional foods online. More than a sixth bought these products more than three times. Only a fifth understood that trading medicines online was not legal in Vietnam. The knowledge scores of Vietnamese people were low, while their attitude scores were reasonably high. The main factors associated with their knowledge and/or attitudes towards purchasing medicines/functional foods online included the frequency of Internet use, seeking health information online for self-diagnosis and self-medication, feeling satisfied with previous experience, having at least one chronic disease, residence, and education level.

In recent years, the development of illegal (rogue/unapproved) online pharmacies selling counterfeit and substandard medications has been an intractable problem globally [30, 31]. In several previous studies, legitimate pharmacies only constituted low proportions of Internet pharmacies [32, 33]. Numerous issues of illegitimate online pharmacies were reported, including not providing any contact details, not offering pharmacist consultations, not requiring prescriptions, not limiting the purchasable quantity, lacking medication information or drug-related warnings, selling substandard and falsified products, and inappropriate packaging and labeling [34–36]. In addition, in our study, a seventh of the participants purchased medicines/functional foods from foreign sources on the Internet. Obviously, trading medicines online can go beyond the scope of a country, causing many difficulties and challenges in management. Hence, it is necessary to enhance people’s knowledge about the online purchase of these products.

Many Vietnamese people had a low knowledge level of online medication purchasing. Nearly two-thirds (63.2%) were aware that medicines/functional foods could be purchased on the Internet, lower than the results of studies in Hungary (82.7%, 2018) [16] and India (83.2%) [18], but far higher than the findings of studies in Saudi Arabia (23.1%, 2013–2014) [15] and Nigeria (28.2%, 2018) [23]. In India, 51.9% of people consulted their doctors before purchasing medicines online [18]. In this study, most Vietnamese people agreed that it is necessary to consult healthcare professionals before buying medicines on the Internet. This can contribute to lowering inappropriate behaviors and irrational medicine use. Besides, it is notable that up to 2023, in Vietnam, no legal documents or laws involving online pharmacies have been promulgated. However, only a fifth of participants knew that trading medicines on the Internet was not legal in Vietnam. Merchandisers in illegal online pharmacies can take advantage of the inadequate perception of the public to sell products, including substandard and counterfeit medications.

Albeit having a low level of knowledge about purchasing medicines/functional foods online, Vietnamese people expressed positive attitudes towards its potential benefits. Several main advantages that many Vietnamese people selected included convenience (87.1%), freedom from location (84.8%), the ability to order and buy products after the opening hours (84.7%), and the ease of checking products’ availability (83.6%), in line with the results of some following studies. In Hungary, convenience and the ability to purchase products without going to conventional pharmacies and after opening hours were the main benefits of online drug shopping selected by patients [16]. In Nigeria, pharmacy customers thought that online pharmacies could have the following benefits: convenience (97.9%), offering greater confidentiality (93.2%), and doorstep delivery (89.0%) [23]. For Indian people, the main benefits of buying medicines online included differences in prices, being able to purchase medicines unavailable in the market, available for 24 h, and being more convenient [18]. In Saudi Arabia, the main motivational factors for purchasing medicinal products from the Internet were the wide variety of products (76.6%), convenience (76.0%), and lower cost (70.0%) [14]. A multinational study in 2015 showed that the main reasons for buying lifestyle products online were convenience, low prices, ease of buying, and quick/time saving [20]. The differences in the results among studies can be explained by the differences in location, studying time, and the sample. Understanding and having positive attitudes towards the benefits of online shopping can contribute to promoting online purchasing behaviors among people.

Besides the benefits mentioned above, there are several potential drawbacks to purchasing medicines/functional foods online. The quality of products sold on the Internet was the issue that Vietnamese people were concerned about the most, in line with Indian people [18]. Findings from a multinational study with the participation of many lifestyle product buyers showed that 62.8% were aware of counterfeit products sold online. About 11.9% had experience with at least one counterfeit lifestyle product [20]. In Romania, only 19.0% of people were aware that medicines bought online could be of inferior quality [11]. Besides merchandise quality, people in Saudi Arabia also felt worried about the easy access of people under 18 years old without restrictions and the lack of supervision of healthcare professionals [14]. Hungarians were concerned about medicine abuse, getting products they did not need or exacerbating their illnesses, and not receiving proper information about product usage [16]. The difficulty in distinguishing between legal and illegal online pharmacies was an additional problem when purchasing health products via the Internet of numerous people in Vietnam and Saudi Arabia [14]. In Vietnam, the Ministry of Health and relevant authorities are drafting legal documents and will enact laws on online pharmacies and trading medicines on the Internet in the forthcoming years. After that, ways to recognize licensed online pharmacies must be widely propagated and disseminated in the community (such as using logos or having a reliable website containing all direct links to the websites of legitimate online pharmacies). Furthermore, with a high proportion of people intending to purchase medicines/functional foods online in the future, it is essential to enhance their knowledge.

The main factors associated with Vietnamese people’s knowledge and/or attitudes towards purchasing medicines/functional foods online included having at least one chronic disease, usually using the Internet, self-diagnosis/self-medication based on health information from the Internet, feeling satisfied with previous online purchasing experience, residence, and education level. People coming down with chronic diseases usually have to take these products daily. Of these people, the proportions of those seeking health information on the Internet for self-diagnosis and self-medication were higher than the figures for those not having a chronic disease. This can explain why people with at least one chronic disease had good knowledge and positive attitudes toward purchasing these products online. An exciting result of this study was that people with a university level of education had lower attitude scores when compared with other groups. The first reason was that the proportion of people who purchased medicines/functional foods online among the former (69.2%) was higher than that of the latter (44.7%). Of buyers, the proportion of people feeling satisfied with previous purchasing experience among university graduates (35.9%) was lower than that of other groups (46.3%). Besides, the low proportion of university graduates working in the medical field can be a rationale behind their low attitude scores. Individuals usually using the Internet also had higher knowledge and more positive attitudes towards this online purchase. The Ministry of Health and relevant authorities can use social networks and online platforms to disseminate reliable information involving online pharmacies and online trading to the public.

In this study, we focused on evaluating the knowledge and attitudes of Vietnamese people regarding purchasing medicines/functional foods on the Internet. There were several other notable findings. First, a majority of Vietnamese people were in the habit of searching for health information on the Internet, which could give rise to self-diagnosis and self-medication, similar to the Romanians [11]. In Germany, Internet users also usually sought information about pharmacies, symptoms, treatment remedies, or ways to live a healthy life on the Internet [2]. In Saudi Arabia, medical and health-related information shared on social networks influenced decisions regarding people’s health care [37]. Some future studies can be conducted to analyze health information-seeking behaviors and their influences on Vietnamese citizens. In addition, this study recorded a high proportion of Vietnamese people purchasing medicines and functional foods in the past year (52.8%), higher than the results of studies in Saudi Arabia (36.5%, 2020) [14], United Arab Emirates (31.2%, 2020) [13], and Hungary (4.2%, 2018) [16]. One rationale behind the high proportion in Vietnam was the time of data collection. We collected data for the past year (mainly for the year 2022) when two severe outbreaks of the COVID-19 pandemic occurred in Vietnam (from February to May 2022 and in August 2022), with tens of thousands of new COVID-19 patients confirmed per day. People were restricted from going outside their houses and online shopping was a perfect choice in the context of social distance and self-isolation. In Vietnam, people usually buy medicines and other health products from public healthcare facilities and private pharmacies. The low availability of essential medicines in public and private sectors, reported in several studies [38, 39], can explain why people used the Internet to seek and purchase medical products. Of Vietnamese buyers, only 41.8% felt satisfied, far lower than the result of a study in Saudi Arabia (80.8%) [14]. In this study, we only mentioned whether or not Vietnamese people purchased medicines/functional foods on the Internet and their level of satisfaction. Further studies should be conducted to analyze online buying activities, the behaviors and intentions of purchasing these products on the Internet, and their associated factors among Vietnamese people.

This is the first study conducted to investigate Vietnamese people’s knowledge, attitudes, and experience in purchasing medicines/functional foods online with a large sample size. Another strength is using the Bayesian Model Averaging method to select variables for the multivariate regression models. However, this study also has several limitations. First and foremost, the causal relationship between independent variables and people’s knowledge and attitudes towards purchasing medicines/functional foods on the Internet cannot be identified by reason of being a cross-sectional study. In the future, more robust study designs, such as longitudinal and experimental studies, can be used instead. Second, in this study, we did not assess the content and construct validity of the questionnaire. A short list of questions may not wholly and comprehensively reflect participants’ knowledge and attitudes towards purchasing medicines/functional foods online. Third, we used convenience and snowballing sampling methods to recruit participants. This could lower the generalization and extrapolation of findings. The sample also may not be representative of the general population because of the high percentages of several subgroups (such as people under 45 years old and those living in urban areas). Lastly, collecting data through a self-report questionnaire can result in recall bias. This weakness can be lowered by minimizing time lapses between past events and the time of data collection, directly interviewing the participants, or using prospective study designs.

## Conclusion

Vietnamese people’s knowledge was low, while their attitudes were relatively positive. With a high proportion of people intending to purchase medicines and functional foods online in the future, enhancing their knowledge is paramount. In the forthcoming years, when the Ministry of Health and relevant authorities publish legal documents and enact laws involving online pharmacies and trading medicines on the Internet, ways to recognize licensed online pharmacies must be widely propagated and disseminated in the community.

## Electronic supplementary material

Below is the link to the electronic supplementary material.

Supplementary Material 1

Supplementary Material 2

## Data Availability

The datasets used and/or analyzed during the current study are available from the corresponding author upon reasonable request.

## Abbreviations

95%CI: 95% Confidence Interval
COVID-19: Coronavirus disease 2019
ICC: Intraclass correlation coefficient
KAP: Knowledge, attitudes, practices
US: The United States
VIF: Variance inflation factor
