# Supplementary material for: Purchasing medicines and functional foods on the internet: a cross-sectional study investigating the knowledge, attitudes, and experience of Vietnamese people in 2023
Source: BMC Public Health. 2024 Sep 27;24:2619. doi: 10.1186/s12889-024-20103-w (PMC11438113; doi:10.1186/s12889-024-20103-w)
Supplement: Supplementary file 1 — Supplementary Material 1 [file 12889_2024_20103_MOESM1_ESM.pdf]

Supplementary Material 1. THE QUESTIONNAIRE

Dear participants. The research team from the Hanoi University of Pharmacy and the Phenikaa University is conducting a study to survey Vietnamese people's knowledge, attitudes, and experience in online purchasing medicines and functional foods (dietary supplements) in 2023. The time for answering questions is about 10 to 15 minutes. All collected information is only used for studying. We hope that you will answer questions fully and honestly. The identity of participants will be kept confidential. We truly appreciate your participation and contribution to our research.

Do you agree to participate in this survey?

☐ Yes ☐ No

1. PERSONAL INFORMATION

No	Question	Answer
1	Year of birth	....
2	Sex	☐ Male ☐ Female
3	Residence (Region)	☐ Northern ☐ Central ☐ Southern
4	Your place (Area)	☐ Urban ☐ Rural
5	Highest level of education	☐ Illiterate ☐ Primary school<br>☐ Secondary school ☐ High school<br>☐ Middle/Intermediate ☐ College<br>☐ University<br>☐ Post-university (Master, Ph.D...)
6	Marital status	☐ Unmarried ☐ Married
7	Working/studying	☐ Healthcare ☐ Non-healthcare
8	Occupation	☐ Studying (student)<br>☐ Unemployed/not working<br>☐ Blue-collar (farmer, worker...)<br>☐ White-collar (doctor, teacher...)<br>☐ Retired<br>☐ Others: ....
9	Average monthly income, allowance, or retirement pension<br>(*unit: million Vietnam dongs*)	☐ No income ☐ < 3<br>☐ 3 to < 6 ☐ 6 to < 9<br>☐ 9 to < 12 ☐ 12 to < 15<br>☐ 15 to < 18 ☐ 18 or above
10	Do you have a health insurance card?	☐ Yes ☐ No
11	Are you contracting any chronic diseases?	☐ Yes ☐ No
12	Name of your chronic disease(s)<br>(if possible)	....<br>..

2. KNOWLEDGE AND ATTITUDES TOWARDS PURCHASING MEDICINES AND FUNCTIONAL FOODS ONLINE

Medicines are preparations/products containing pharmaceutical substances or herbal ingredients for use in humans for the purpose of disease prevention, diagnosis, treatment, alleviation, and adjustment of

physiological functions of the human body, including chemical medicines, herbal medicines, traditional medicines, vaccines, and biological products.

Dietary supplements (functional foods) are foods used to support the function of parts of the human body, have nutritional effects, make the body comfortable, increase immunization, and reduce the risk of contracting diseases. *To distinguish from medicines, on the packaging of dietary supplements, there are the words "Dietary supplement/functional food" and "This product is not a medicine and does not have the effects of replacing medicines".*

Online shopping is a form of e-commerce that allows consumers to purchase goods or services directly from a seller over the Internet using a web browser or mobile app. Goods are distributed through electronic exchanges, websites, and social networks...

	**Statement**	**Totally disagree**	**Disagree**	**Neutral**	**Agree**	**Totally agree**	**Reference**
1	Medicines and functional foods can be purchased on the Internet (online).	☐	☐	☐	☐	☐	[1, 2, 5, 6, 8, 12]
2	There are other reputable sources to buy medicines and functional foods besides pharmacies in the hospitals and the community.	☐	☐	☐	☐	☐	[8, 11, 12]
3	It is necessary to check the information (such as qualification and reputation) of the sellers/providers before online buying.	☐	☐	☐	☐	☐	[3, 6]
4	Nearly every medicine and functional food can be purchased on the Internet.	☐	☐	☐	☐	☐	[11, 12]
5	It is necessary to consult with healthcare professionals (such as doctors and pharmacists) before buying medicines online, especially prescription-only medicines.	☐	☐	☐	☐	☐	[1]
6	Purchasing prescription-only medicines online without a prescription is legal and appropriate.	☐	☐	☐	☐	☐	[14]
7	Trading medicines online can go beyond the scope of a country, causing many difficulties and challenges in management.	☐	☐	☐	☐	☐	[2, 3, 6]
8	Selling medicines online is legal in Vietnam.	☐	☐	☐	☐	☐	[14, 15]
	**Potential advantages of purchasing medicines and functional foods on the Internet**						
1	Wide range of products (can buy products unavailable or not sold at nearby local hospitals or community pharmacies)	☐	☐	☐	☐	☐	[2, 4, 5, 7, 8, 10]
2	In comparison with buying products in community pharmacies, I can get more information when buying online	☐	☐	☐	☐	☐	[4, 6, 10, 11, 12]
3	Affordable, lower prices, many offers or discounts	☐	☐	☐	☐	☐	[2, 4, 6, 7, 9, 10]

	Statement	Totally disagree	Disagree	Neutral	Agree	Totally agree	Reference
4	When buying online, it is faster and easier to compare the information and prices of products than when directly buying in pharmacies	☐	☐	☐	☐	☐	[6, 11]
5	Able to order and buy products after opening hours (available 24/7)	☐	☐	☐	☐	☐	[2, 6, 10, 11]
6	More privacy and anonymity	☐	☐	☐	☐	☐	[2, 4, 9, 10, 13]
7	Convenience (for example, doorstep delivery; in conditions of bad weather, pandemics, or epidemics; elimination of barriers for people with disabilities and senior citizens)	☐	☐	☐	☐	☐	[2, 4, 5, 6, 8, 9, 10, 13]
8	Easily check the availability of products (reduce the time spent traveling between pharmacies to check)	☐	☐	☐	☐	☐	[6, 12]
9	Freedom from location (can purchase products from other countries)	☐	☐	☐	☐	☐	[2, 12]
10	No waiting time or queuing at pharmacies	☐	☐	☐	☐	☐	[5, 8]
	**Potential disadvantages of purchasing medicines and functional foods on the Internet**						
11	Increasing the risks of polypharmacy and drug interactions	☐	☐	☐	☐	☐	[2]
12	Can purchase expired, counterfeit, and/or substandard products	☐	☐	☐	☐	☐	[2, 4, 6, 8, 9, 13]
13	Information about products on the Internet may be inaccurate	☐	☐	☐	☐	☐	[4, 6, 11]
14	Long delivery time	☐	☐	☐	☐	☐	[2, 13]
15	Increasing the risks of personal information leakage and money transactions	☐	☐	☐	☐	☐	[9]
16	Not getting the right products (mistakes in packing or delivery)	☐	☐	☐	☐	☐	[4, 6, 10, 11, 13]
17	Lack of supervision of the authorities (such as the seller may not be licensed to sell medicines)	☐	☐	☐	☐	☐	[4, 8, 9]
18	Difficult to choose the appropriate product due to the great number of products on the Internet	☐	☐	☐	☐	☐	[6, 11, 12]
19	Products can be stored/preserved under substandard conditions	☐	☐	☐	☐	☐	[2, 5]
20	The sellers may not be precisely identified (names, qualifications...)	☐	☐	☐	☐	☐	[3, 12]

	Statement	Totally disagree	Disagree	Neutral	Agree	Totally agree	Reference
21	Increasing the risks of drug abuse, self-medication, and treatment non-adherence	☐	☐	☐	☐	☐	[2, 6, 11, 12]
22	People under 18 years old can purchase medications without restrictions	☐	☐	☐	☐	☐	[4]
23	Difficult to distinguish between legal (registered) online pharmacies and illegal (unlicensed) commercial websites	☐	☐	☐	☐	☐	[2, 4, 5]

3. INTERNET USE AND ONLINE PURCHASE OF MEDICINES AND FUNCTIONAL FOODS (in the past year)

	In the past year...		Reference
1	Frequency of Internet use	☐ Rarely ☐ Sometimes<br>☐ Usually	
2	Frequency of online shopping<br>(for any products or services)	☐ Never<br>☐ Rarely (1-3 times/month)<br>☐ Sometimes (1-2 times/week)<br>☐ Regularly (>3 times/week)<br>☐ Others:	
3	Using the Internet to seek health information	☐ Yes ☐ No	
4	Self-diagnosis without visiting a doctor based on health information from the Internet	☐ Yes ☐ No	
5	Self-medication without consulting a pharmacist or doctor based on information from the Internet	☐ Yes ☐ No	
6	Have you ever purchased medicines online?	☐ Yes ☐ No	[1, 2, 5, 6]
7	Have you ever purchased functional foods online?	☐ Yes ☐ No	[2, 6]
8	Have you ever bought medicines or functional foods online from foreign sources (such as international websites)	☐ Yes ☐ No	[1, 5]
9	Names of medicines/functional foods you have purchased from the Internet	....<br>..	[3, 4, 10]
10	Number of times purchasing medicines and/or functional foods online	About .... times	[5]
11	Are you satisfied with your previous experiences in purchasing medicines and functional foods on the Internet?	☐ Completely unsatisfied<br>☐ Unsatisfied<br>☐ Normal/Neutral<br>☐ Satisfied<br>☐ Totally satisfied<br>☐ Never shopped online	[4, 5]

12	Reasons for your satisfied or dissatisfied experience	....<br>..	[4, 5]

Thank you for your participation in this research.

References

1. Pal S, Laszlo K, Andras F, Gabriel H, Hajnal F, Adriana C, et al. Attitude Of Patients And Customers Regarding Purchasing Drugs Online. Farmacia. 2015;63(1):93–98.

2. Fittler A, Lankó E, Brachmann B, Botz L. Behaviour analysis of patients who purchase medicines on the internet: can hospital pharmacists facilitate online medication safety? European Journal of Hospital Pharmacy 2013;20:8-12.

3. Jairoun AA, Al-Hemyari SS, Abdulla NM, El-Dahiyat F, Jairoun M, Al-Tamimi SK, et al. Online medication purchasing during the Covid-19 pandemic: potential risks to patient safety and the urgent need to develop more rigorous controls for purchasing online medications, a pilot study from the United Arab Emirates. J Pharm Policy Pract. 2021;14(1):38. doi: 10.1186/s40545-021-00320-z. Erratum in: J Pharm Policy Pract 2021;14(1):44.

4. Alwhaibi M, Asser WM, A Al Aloola N, Alsalem N, Almomen A, Alhawassi TM. Evaluating the frequency, consumers' motivation and perception of online medicinal, herbal, and health products purchase safety in Saudi Arabia. Saudi Pharm J. 2021;29(2):166-172. doi: 10.1016/j.jsps.2020.12.017.

5. Abanmy N. The extent of use of online pharmacies in Saudi Arabia. Saudi Pharm J. 2017;25(6):891-899. doi: 10.1016/j.jsps.2017.02.001.

6. Fittler A, Vida RG, Káplár M, Botz L. Consumers Turning to the Internet Pharmacy Market: Cross-Sectional Study on the Frequency and Attitudes of Hungarian Patients Purchasing Medications Online. J Med Internet Res. 2018;20(8):e11115. doi: 10.2196/11115.

7. Bowman C, Family H, Agius-Muscat H, Cordina M, Sutton J. Consumer internet purchasing of medicines using a population sample: A mixed methodology approach. Res Social Adm Pharm. 2020;16(6):819-827. doi: 10.1016/j.sapharm.2019.09.056.

8. Bansal S, Kaur H, Mahendiratta S, Sarma P, Kumar S, Sharma AR, et al. A preliminary study to evaluate the behavior of Indian population toward E-pharmacy. Indian J Pharmacol. 2022;54(2):131-137. doi: 10.4103/ijp.ijp_836_21.

9. Almomani H, Patel N, Donyai P. News Media Coverage of the Problem of Purchasing Fake Prescription Medicines on the Internet: Thematic Analysis. JMIR Form Res. 2023;7:e45147. doi: 10.2196/45147.

10. Assi S, Thomas J, Haffar M, Osselton D. Exploring Consumer and Patient Knowledge, Behavior, and Attitude Toward Medicinal and Lifestyle Products Purchased From the Internet: A Web-Based Survey. JMIR Public Health Surveill. 2016;2(2):e34. doi: 10.2196/publichealth.5390.

11. Fittler A, Ambrus T, Serefko A, Smejkalová L, Kijewska A, Szopa A, et al. Attitudes and behaviors regarding online pharmacies in the aftermath of COVID-19 pandemic: At the tipping point towards the new normal. Front Pharmacol. 2022;13:1070473. doi: 10.3389/fphar.2022.1070473.

12. Lobuteva L, Lobuteva A, Zakharova O, Kartashova O, Kocheva N. The modern Russian pharmaceutical market: consumer attitudes towards distance retailing of medicines. BMC Health Serv Res. 2022;22(1):582. doi: 10.1186/s12913-022-07991-7.

13. Ndem E, Udoh A, Awofisayo O, Bafor E. Consumer and Community Pharmacists' Perceptions of Online Pharmacy Services in Uyo Metropolis, Nigeria. Innov Pharm. 2019;10(3):10.24926/iip.v10i3.1774. doi: 10.24926/iip.v10i3.1774.

14. The Vietnam National Assembly. Law on Pharmacy, No. 105/2016/QH13. 2016.

15. TuoiTre Online. Ho Chi Minh City Department of Health: Selling drugs via social networks is a violation that will be corrected and handled. 2022. Available from: https://tuoitre.vn/so-y-te-tp-hcm-ban-thuoc-qua-mang-xa-hoi-la-vi-pham-se-chan-chinh-xu-ly-20220519181204089.htm
