# Supplementary material for: Purchasing medicines and functional foods on the internet: a cross-sectional study investigating the knowledge, attitudes, and experience of Vietnamese people in 2023
Source: BMC Public Health. 2024 Sep 27;24:2619. doi: 10.1186/s12889-024-20103-w (PMC11438113; doi:10.1186/s12889-024-20103-w)
Supplement: Supplementary file 2 — Supplementary Material 2 [file 12889_2024_20103_MOESM2_ESM.pdf]

Table S1. Factors associated with the knowledge and attitudes of the participants regarding purchasing medicines and functional foods on the Internet (univariate linear regression analyses)

No	Independent variables		Knowledge		Attitude	
			coef	p-value	coef	p-value
1	Age (years old) (continuous variable)		-0.012	0.063	0.036	0.317
2	Sex (ref: Female)	Male	0.156	0.325	0.368	0.676
3	Region (ref: Central and Southern)	Northern	-0.716	<0.001	-1.654	0.052
4	Area (ref: Rural)	Urban	-0.186	0.325	1.391	0.187
5	Marital status (ref: Married)	Unmarried	0.448	0.003	1.139	0.175
6	Highest level of education (ref: College or intermediate)	Secondary or lower	-0.222	0.636	-5.598	0.033
		High school	0.580	0.021	-0.316	0.822
		University	0.004	0.987	-3.428	0.019
		Post-university	1.744	<0.001	4.260	0.077
7	Occupation (ref: Healthcare)	Non-healthcare, students, retired	0.000	1.000	3.719	0.026
8	Income/allowance/retirement pension (unit: mVND*) (ref: 12 to <18)	<6	-0.038	0.870	-3.882	0.003
		6 to <12	-0.011	0.968	-2.583	0.078
		18 or more	-0.365	0.190	-5.354	<0.001
9	Having at least one chronic disease (ref: No)		0.534	0.005	5.787	<0.001
10	Frequency of Internet use (ref: Rarely)	Sometimes	0.592	0.085	6.313	0.001
		Usually	1.357	<0.001	7.709	<0.001
11	Online shopping (ref: Rarely or never)	Sometimes	0.430	0.009	1.183	0.200
		Usually	1.099	<0.001	5.198	<0.001
12	Using the Internet for self-diagnosis (ref: No)		0.815	<0.001	5.715	<0.001
13	Using the Internet for self-medication (ref: No)		1.295	<0.001	6.405	<0.001
14	Purchased medicines and/or functional foods on the Internet in the past year (ref: No)		0.463	0.002	0.809	0.336
15	Level of satisfaction (ref: Unsatisfied)	Never	-0.376	0.390	-0.936	0.708
		Neutral	-0.753	0.091	-3.228	0.206
		Satisfied	1.160	0.010	3.772	0.144

No	Independent variables	Knowledge		Attitude	
		coef	p-value	coef	p-value
16	Times of purchasing medicines and functional foods online in the past year (continuous variable)	0.016	0.362	0.025	0.801
17	Knowledge score (continuous variable)			2.914	<0.001
coef: coefficient, ref: reference. *: 1 mVND (million Vietnam dongs) = 49.194 US$.					
